# m^6^A mRNA Methylation Regulates Epithelial Innate Antimicrobial Defense Against Cryptosporidial Infection

**DOI:** 10.3389/fimmu.2021.705232

**Published:** 2021-07-06

**Authors:** Zijie Xia, Jihao Xu, Eugene Lu, Wei He, Silu Deng, Ai-Yu Gong, Juliane Strass-Soukup, Gislaine A. Martins, Guoqing Lu, Xian-Ming Chen

**Affiliations:** ^1^ Department of Medical Microbiology and Immunology, Creighton University School of Medicine, Omaha, NE, United States; ^2^ Department of Biology, School of Interdisciplinary Informatics, University of Nebraska at Omaha, Omaha, NE, United States; ^3^ Department of Microbial Pathogens and Immunity, Rush University Medical Center, Chicago, IL, United States; ^4^ Department of Chemistry, Creighton University College of Arts & Sciences, Omaha, NE, United States; ^5^ Department of Medicine and Biomedical Sciences, Research Division of Immunology Cedars-Sinai Medical Center, David Geffen School of Medicine at UCLA, Los Angeles, CA, United States

**Keywords:** m^6^A, *Cryptosporidium*, intestinal epithelium, defense, ALKBH5, RNA stability, Irgm2, Igtp

## Abstract

Increasing evidence supports that N6-methyladenosine (m^6^A) mRNA modification may play an important role in regulating immune responses. Intestinal epithelial cells orchestrate gastrointestinal mucosal innate defense to microbial infection, but underlying mechanisms are still not fully understood. In this study, we present data demonstrating significant alterations in the topology of host m^6^A mRNA methylome in intestinal epithelial cells following infection by *Cryptosporidium parvum*, a coccidian parasite that infects the gastrointestinal epithelium and causes a self-limited disease in immunocompetent individuals but a life-threatening diarrheal disease in AIDS patients. Altered m^6^A methylation in mRNAs in intestinal epithelial cells following *C. parvum* infection is associated with downregulation of alpha-ketoglutarate-dependent dioxygenase alkB homolog 5 and the fat mass and obesity-associated protein with the involvement of NF-кB signaling. Functionally, m^6^A methylation statuses influence intestinal epithelial innate defense against *C. parvum* infection. Specifically, expression levels of immune-related genes, such as the immunity-related GTPase family M member 2 and interferon gamma induced GTPase, are increased in infected cells with a decreased m^6^A mRNA methylation. Our data support that intestinal epithelial cells display significant alterations in the topology of their m^6^A mRNA methylome in response to *C. parvum* infection with the involvement of activation of the NF-кB signaling pathway, a process that modulates expression of specific immune-related genes and contributes to fine regulation of epithelial antimicrobial defense.

## Introduction

Increasing evidence supports that RNA methylation is a widespread phenomenon and a critical regulator of gene expression (1, 2). The most prevalent RNA methylation, N6-methyladenosine (m^6^A), is a reversible RNA post-transcriptional modification and occurs in approximately 25% of transcripts at the genome-wide level (1). RNA m^6^A methylation regulates RNA splicing, translocation, stability, and translation into protein (3–6). Dynamic regulation of the m^6^A epi-transcriptome is involved in diverse cellular functions, including heat shock, DNA damage, cancer, stem cell differentiation, circadian rhythm, spermatogenesis and oogenesis, response to interferon-γ, and viral infections (2, 3, 7). m^6^A dynamics and functions are executed by three groups of proteins: methyltransferases or “writers”, demethylases or “erasers”, and m^6^A-binding proteins or “readers” (2, 3, 7). In most cell types, m^6^A methylation is catalyzed by the methyltransferase complex consisting of the methyltransferase-like 3 (METTL3) and METTL14, as well as their cofactors (3, 7). The erasers include the fat mass and obesity-associated protein (FTO) and alpha-ketoglutarate-dependent dioxygenase alkB homolog 3 (ALKBH3) and ALKBH5 (2, 3, 7). Recent studies demonstrate that m^6^A methylation may play an important role in regulating immune responses (8, 9). It has been associated with numerous physiological and pathological phenomena, including obesity, immunoregulation, yeast meiosis, plant development, and carcinogenesis (2, 10). Specifically, m^6^A methylation has been recognized as crucial regulator in T cell homeostasis, inflammation, type I interferon production, and the immune response to bacterial or viral infection (3, 8–11). Selectively altered m^6^A levels along with other types of immunotherapies may be efficient management strategies in a variety of immunological diseases.

Epithelial cells along the mucosal surface provide the front line of defense against luminal pathogen infection in the gastrointestinal tract and are an important component of gastrointestinal mucosal immunity (12). Intestinal epithelial cells generate various types of barriers to protect the intestinal mucosa from commensal microbes or invading pathogenic microorganisms. Upon microbial challenge, gastrointestinal epithelial cells quickly initiate a series of innate immune reactions, including production of antimicrobial molecules and release of inflammatory chemokines/cytokines. These chemokines/cytokines of epithelial cell origin may mobilize and activate immune effector cells to the infection sites (13). Therefore, intestinal epithelial cells not only create mucosal barriers to ‘segregate’ gut microbes and gut immune cells but also sense signals from both populations and secrete humoral factors to ‘mediate’ the balance between both populations (14). Failure to maintain the complex functional and anatomical features of the intestinal epithelium reduces the antimicrobial, immunoregulatory and regenerative ability of the epithelial barrier and might allow translocation of commensal bacteria from the intestinal lumen to the subepithelial tissue (13, 14). Although much is known about the active role of epithelial innate immune stimulation in antimicrobial host defense and host–microbial homeostasis, how intestinal epithelial cells orchestrate gastrointestinal mucosal defense and homeostasis is still not fully understood.

*Cryptosporidium spp*, a coccidian parasite and an NIAID Category B priority pathogen, infects the gastrointestinal epithelium and causes a self-limited disease in immunocompetent individuals but a life-threatening diarrheal disease in AIDS patients (15–17). After rotavirus, *Cryptosporidium* is the most common pathogen responsible for moderate-to-severe diarrhea in children younger than 2 years (18). The majority of human cryptosporidial infections are caused by two species: *C. parvum* and *C. hominis* (15). *C. parvum* attaches to the apical membrane surface of intestinal epithelial cells (mainly enterocytes) and forms an intracellular but extra-cytoplasmic vacuole in which the organism remains (15). Thus, *C. parvum* is classified as a “minimally invasive” mucosal pathogen (15) and innate epithelial defense is critical to the host’s defense against *C. parvum* infection (19). In this study, we present data demonstrating significant alterations in the topology of host m^6^A mRNA methylome in intestinal epithelial cells following *C. parvum* infection. *C. parvum* infection promotes m^6^A mRNA methylation in intestinal epithelial cells through downregulation of Alkbh5 with the involvement of NF-кB signaling. Functionally, m^6^A methylation statuses influence intestinal epithelial anti-*C. parvum* defense. Specifically, expression levels of immune-related genes, such as the immunity-related GTPase family M member 2 (*Irgm2*) and interferon gamma induced GTPase (*Igtp*, also called as *Irgm3* in mice), are increased in infected cells with a decreased m^6^A mRNA methylation. Our data support that intestinal epithelial cells display significant alterations in the topology of their m^6^A mRNA methylome in response to *C. parvum* infection with the involvement of activation of the NF-кB signaling pathway, a process that modulates expression of specific immune-related genes and contributes to fine regulation of epithelial antimicrobial defense.

## Materials and Methods

### *C. parvum* and Cell Lines

*C. parvum* oocysts of the Iowa strain were purchased from a commercial source (Bunch Grass Farm, Deary, ID). The mouse intestinal epithelial cell line (IEC4.1) was received as a kind gift from Dr. Pingchang Yang (McMaster University, Hamilton, Canada). The HCT-8 cells were human intestinal epithelial cells from ATCC (Manassas, Virginia). The BV2 mouse microglia cells and RAW264.7 mouse macrophage cells were obtained from ATCC. Culture media were supplied with 10% FBS (Ambion, Austin, Texas) and antibiotics (100 IU/ml of penicillin and 100 µg/ml of streptomycin).

### Infection Models and Infection Assays

Models of intestinal cryptosporidiosis using intestinal epithelial cell lines and enteroids were employed; infection was done with a 1:1 ratio between *C. parvum* oocysts and host cells as previously described (20–22). Intestinal epithelium and enteroids were isolated and cultured as previously described (22). Briefly, small intestines were opened longitudinally and washed with ice-cold Ca2^+^ and Mg2^+^ free PBS, then were cut into 1-2 mm fragments and washed with ice-cold Ca2^+^ and Mg2^+^ free PBS 3 times. The cut fragments were incubated in ice-cold 2 mM PBS/EDTA at 4°C for 30 min with gentle rotation followed by vigorous shake until the PBS solution was mostly opaque with dislodged crypt and villus particles. Large tissue fragments were removed through a 100-µm cell strainer (Becton-Dickinson Bioscience, Franklin Lakes, NJ). The pass through was centrifuged 150g for 5 min at 4°C and the pellet was collected as the intestinal epithelium. The 2D monolayers were derived from 3D enteroids as previously reported and cultured for *C. parvum* infection for 24-48 h. A well-developed infection model of cryptosporidiosis in neonatal mice was used for *in vivo* experiments (23, 24). Mice at the age of 5 days after birth received *C. parvum* oocysts by oral gavage (10^5^ oocysts per mice). Mice receiving vehicle (PBS) by oral gavage were used as control. The C57BL/6N mice (from the Jackson Lab, Bar Harbor, Maine) were used for this study, in accordance with procedures (protocol number #0959) approved by the Institutional Animal Care and Use Committee of Creighton University. Real-time PCR, immunofluorescence microscopy, and immunohistochemistry were used to assay *C. parvum* infection as previously reported (25, 26).

### PCR

For quantitative analysis of RNA expression, comparative real-time PCR was performed as previous reported (20, 22) using the SYBR Green PCR Master Mix (Applied Biosystems, Carlsbad, CA). The sequences for all the primers described above are listed in **Table S3**.

### siRNAs

The mouse Alkbh5 siRNA (#sc-141022) and human ALKBH5 siRNA (#sc-93856) were purchased from the Santa Cruz Biotechnology. Custom-designed RNA oligos against Alkbh5 and a scrambled RNA were synthesized by Exiqon and transfected into cells (at a final concentration of 10 pmol for 48 h) with Lipofectamine RNAimax according to the manufacturer’s protocol (Invitrogen). Sequences of siRNAs are: GAAAUGCUAACCGAGCUCAUU (sense) and UGAGCUCGGUUAGCAUUUCUU (antisense) for human ALKBH5 and GAAAUGCUAACCGAGCUCAUU (sense) and UGAGCUCGGUUAGCAUUUCUU (antisense) for mouse Alkbh5. The non-specific scrambled sequence UUCUCCGAACGUGUCACGUUU (sense) and ACGUGACACGUUCGGAGAAUU (antisense) for the control. siRNAs were transfected into IEC4.1 cells with Lipofectamine RNAimax (Invitrogen).

### CRISPR/Cas9 Approach to Generate Stably Transfected Cell Lines

CRISPR/Cas9 was applied to stably knock out or activate the *Alkhb5* gene (NCBI GeneID 268420) and *Fto* gene (NCBI GeneID 26383) to generate stable cell lines. The mouse Alkbh5 CRISPR/Cas9 KO Plasmid (sc-435243), mouse Alkbh5 CRISPR Activation Plasmid (sc-435243-ACT), mouse Fto CRISPR/Cas9 KO Plasmid (sc-424024), and mouse Fto CRISPR Activation Plasmid (sc-424024-ACT) were purchased from the Santa Cruz Biotechnology. The plasmids were transfected to cells with UltraCruz^®^ Transfection Reagent following the manufacturer’s protocol (Santa Cruz Biotechnology, Inc.). Colonies were selected, and Western blot was used to detect Alkbh5 and Fto protein expression. The clones with the expected knockdown and overexpression of Alkbh5 and Fto were further validated by qPCR and Sanger sequencing.

### Western Blot

Protein concentration was determined and subsequently analyzed by Western blot. The following antibodies were used for blotting: anti-Alkbh5 (Cell Signaling, #802835), anti-Fto (Cell Signaling, #45980), anti-Gapdh (Santa Cruz Biotechnology, sc-365062), and anti-β-Actin (Cell Signaling, #8457).

### rRNA Removal and Quality Analysis

Total RNA was isolated from IEC 4.1 cells with TRAZOL Reagent (Invitrogen). Contaminated rRNA was removed by using RiboMinus™ Eukaryote Kit (Invitrogen, #A10837-08). The ribosomal RNA depleted RNA concentration was fragmented using RNA fragmentation reagents (Invitrogen, #AM8740). The RNA fragment was measured by NanoDrop and the quality of RNA was analyzed with Agilent 2100 bioanalyzer.

### m^6^A Dot Blot

The ribosomal RNA depleted RNA concentration of each whole cell lysate was determined and subsequently analyzed by dot-blot. Anti-m^6^A (Synaptic Systems, #202003) was used for blotting. Isolated RNA was first denatured by heating at 95°C for 3 min, followed by chilling on ice rapidly. Two-fold serial dilutions were spotted on an Amersham Hybond-N+ membrane optimized for nucleic acid transfer (GE Healthcare). After UV crosslinking in a Stratagene Stratalinker 2400 UV Crosslinker, the membrane was washed by 1×TBST buffer, blocked with 5% of non-fat milk in TBST, and incubated with anti-m^6^A antibody (1:1,000) overnight at 4°C. After incubating with HRP-conjugated anti-rabbit IgG secondary antibody, the membrane was visualized by ECL Western Blotting Detection Kit (Thermo).

### RNA Stability

RNA stability assay was performed by real-time PCR as previously reported (27); modifications are described in the **Supplemental Experimental Procedures**.

### Luciferase Assay

The promoter region sequence of Alkbh5 or Fto (-2kb~0) was cloned into the pGL3 vector, and plasmids were transfected to IEC4.1 cells with Lipofectamine 2000 following the manufacturer’s protocol (Santa Cruz Biotechnology). Transient transfected cells were harvested with Reporter lysis buffer (Progema). The activity of luciferase was then determined by Luciferase assay system (Progema) as previously reported (28). For specific details, see the **Supplemental Information** and **Table S3**.

### ChIP Analysis

The formaldehyde crosslinking ChIP was performed as described (28–30). ChIP analysis was performed with a commercially available ChIP Assay Kit (Upstate Biotechnologies) in accordance with the manufacturer’s instructions. For specific details, see the **Supplemental Information** and **Table S3**.

### m^6^A RNA Methylation Quantitation Measurement

Total of 200 ng of ribosomal RNA depleted RNA from pretreatment of IEC4.1 or HCT-8 cells were used, and m^6^A quantification was accomplished by using EpiQuik m^6^A Methylation Quantification kit (Colorimetric, Epigentek) according to the manufacturer’s instructions.

### RNA-Seq and m^6^A-RNA Immunoprecipitation (MeRIP-seq) Seq

RNA-seq was accomplished as previously reported (28). Total RNA was isolated from cells with TRIzol Reagent (Invitrogen). 1μg RNA was used to construct libraries with TruSeq Stranded total RNA Library Prep Kit (Illumina, San Diego, CA) and the residual RNA was used for RNA-seq. Sequencing was carried out on Illumina HiSeq 4000 according to the manufacturer’s instructions with single-end 50 bp read length. MeRIP-seq was accomplished as previously reported (31). Ribosomal RNA depleted RNA was isolated, purified by using RiboMinus™ Eukaryote Kit (Invitrogen, #A10837-08) and chemically shredded into ~100 nt fragments by using RNA fragmentation reagents (Invitrogen, #AM8740). RNA fragments (2000 ng) were denatured at 95°C for 3 min and incubated with 20 µl of Magna ChIP Protein A+G Magnetic Beads (Millipore, #2923270) conjugated to anti−m^6^A antibody (2.5 µg, Synaptic Systems, # 202003) or rabbit control IgG (Cell Signaling Technology) in 1X IPP buffer (15 mM NaCl, 10 mM Tris-HCl and 0.1% NP-40) with rotation at 4°C for 4 h. The beads were washed twice with 1X IPP buffer, twice with low−salt buffer (50 mM NaCl, 10 mM Tris-HCl and 0.1% NP-40), twice with high-salt buffer (500 mM NaCl, 10 mM Tris-HCl and 0.1% NP-40) and once with 1X IPP buffer. RNA was eluted from the beads with RLT buffer and purified through Qiagen RNeasy columns (Qiagen, #74104) according to the manufacturer’s recommendation. RNA fragments were purified from the eluates with RNA Clean and Concentrator (Zymo) and used to construct libraries with TruSeq Stranded mRNA Library Prep Kit (Illumina, San Diego, CA). Sequencing was carried out on Illumina HiSeq 4000 according to the manufacturer’s instructions with single-end 50 bp read length.

### Bioinformatics and Statistical Analysis

For specific details about the bioinformatic analysis, see the **Supplemental Experimental Procedures**. All values are given as mean ± S.E. Means of groups were from at least three independent experiments and compared with Student’s t test (unpaired) or the ANOVA test when appropriate. p values < 0.05 were considered statistically significant.

## Results

### Elevated Level of Global mRNA m^6^A Methylation in Intestinal Epithelium Following *Cryptosporidium* Infection

**Figure 1:**
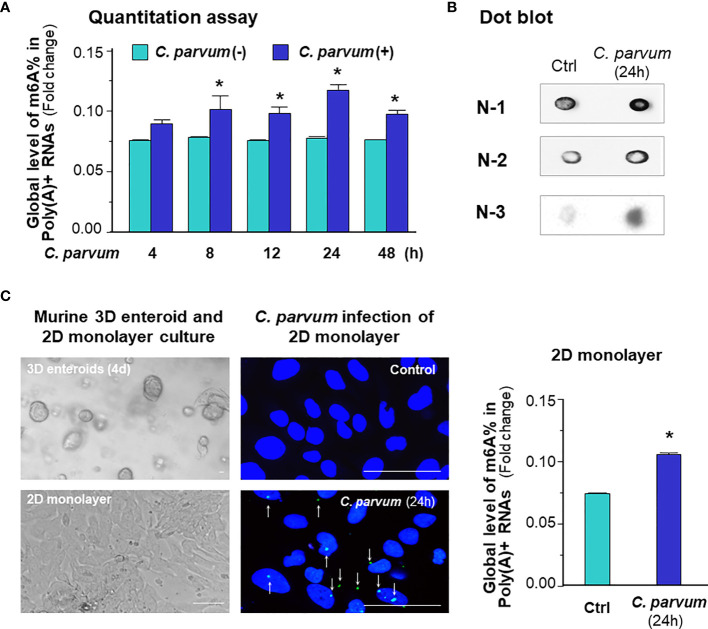
*C. parvum* infection causes a global increase in m^6^A RNA methylation in intestinal epithelial cells. **(A)** Increase of m^6^A RNA methylation in IEC4.1 cells following *C. parvum* infection. Cells were exposed to *C. parvum* for up to 48 h and RNA was collected for m^6^A methylation measurement with the m^6^A RNA methylation quantitation assay. **(B)** Dot blot measurement of m^6^A RNA methylation in IEC4.1 cells following *C. parvum* infection. Cells were exposed to *C. parvum* infection for 24 h and RNA was collected for dot blot with anti-m^6^A. Gel images from three independent experiments are shown. **(C)** Increase of m^6^A RNA methylation in 2D murine intestinal epithelial monolayers following *C. parvum* infection. The crypt units of small intestinal epithelium from neonates of 5 days old were isolated and cultured into 2D monolayers followed by exposure to *C. parvum* infection for 24 h, as shown by phase and immunofluorescence microscopy (*C. parvum* parasites were stained in green as indicated by arrows). Intestinal epithelial 2D monolayers were exposed to *C. parvum* for 24h and RNA was collected for m^6^A methylation measurement with the m^6^A RNA methylation quantitation assay. Bar = 20 μm. Data represent three independent experiments. *p < .05 *vs* the non-infected control.

We first characterized the global mRNA m^6^A status in intestinal epithelial cells following *C. parvum* infection. Using an *in vitro* infection model employing IEC4.1 cells, which are transformed but non-tumorigenic intestinal epithelial cells from neonatal mice (5-7 days old) (32) and received from Dr. Pingchang Yang (McMaster University, Hamilton, Canada), we measured the global m^6^A mRNA methylation levels using the m^6^A RNA methylation quantitation assay and dot blot as previously reported (33, 34). We demonstrated a significant increase in the m^6^A mRNA methylation level in IEC 4.1 cells following *C. parvum* infection as revealed by m^6^A RNA methylation quantitation assay (**Figure 1A**) and dot blot (**Figure 1B**). Using an *ex vivo* infection model employing 2D enteroid monolayers from neonatal mouse ileum (23, 24), we detected an increase of global m^6^A mRNA methylation level in infected intestinal epithelial monolayers (**Figure 1C**). Previous studies indicate that *C. parvum* infection activates NF-кB and IFN-α signaling in infected host cells (25, 28, 29, 35). To define if elevated m^6^A mRNA methylation level in infected cells is due to activation of NF-кB and/or IFN-α signaling, we measured the m^6^A mRNA methylation levels in IEC4.1 cells following stimulation by TNF-α (to activate NF-кB signaling) and IFN-α. Indeed, induction of m^6^A mRNA methylation status was detected in IEC4.1 cells stimulated with TNF-α and IFN-α (**Figure S1**).

### *C. parvum* Infection Promotes m^6^A mRNA Methylation in Intestinal Epithelial Cells Through Downregulation of Alkbh5 and Fto With the Involvement of NF-кB Signaling

**Figure 2:**
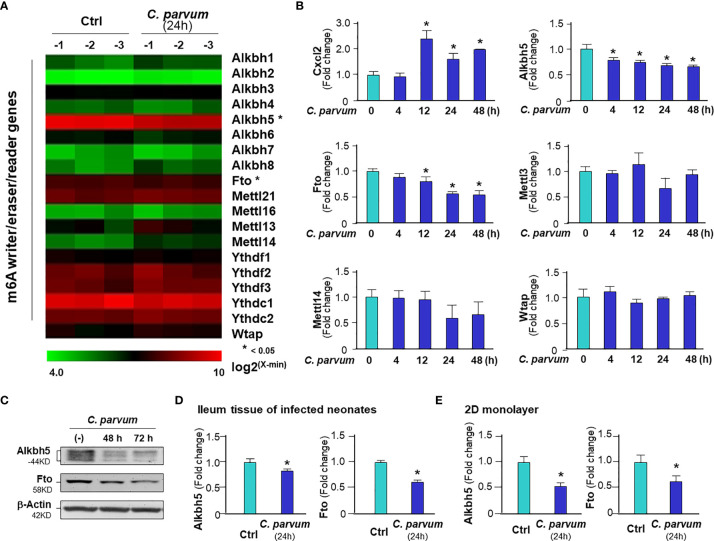
Downregulation of Alkbh5 and Fto in intestinal epithelial cells *C. parvum* infection. **(A)** Heatmaps showing expression profile of key genes involved in the m^6^A RNA methylation machinery in IEC4.1 cells following *C. parvum* infection. Cells were exposed to *C. parvum* infection for 24 h followed by genome-wide array analysis. **(B)** Dynamics of Alkbh5 and Fto downregulation in IEC4.1 cells following *C. parvum* infection. IEC4.1 were exposed to *C. parvum* infection for 4-48 h and RNA expression levels of Alkbh5 and Fto were validated by using real-time quantitative PCR. Expression levels of Cxcl2 (as a positive control), Mettl3, Mettl14 and Wtap were also measured. **(C)** Decreased abundance of Alkbh5 and Fto proteins in IEC4.1 cells following *C. parvum* infection. IEC4.1 were exposed to *C. parvum* infection for 48-72 h and expression levels of Alkbh5 and Fto at the protein level were validated using Western blot. β-Actin was also blotted for internal control. Representative gels were shown. **(D)** Downregulation of Alkbh5 and Fto in murine intestinal epithelium following *C. parvum* infection *in vivo*. Neonates of mice at 5 days of age received *C. parvum* administration by oral gavage and intestinal ileum epithelium were isolated after infection for 24h. Expression levels of expression levels of Alkbh5 and Fto were measured. **(E)** Downregulation of Alkbh5 and Fto in 2D murine intestinal epithelial monolayers following *C. parvum* infection *ex vivo*. Data represent three independent experiments. *p<.05 *vs* the non-infected control.

To explore the underlying mechanism of *C. parvum*-induced m^6^A mRNA methylation in infected intestinal epithelial cells, we analyzed the expression levels of key effectors regulating m^6^A methylation in general, including the major writers, erasers and readers (2, 3, 7). We previously performed a genome-wide transcriptome analysis of *C. parvum*-infected IEC4.1 cells (28). From this dataset, out of the genes coding these key effector molecules, we found out significant decreased RNA expression levels of *Alkbh5* and *Fto*, while others showing no significant changes in their expression levels (**Figure 2A**). We therefore focused on the two genes to test whether decrease of their expression levels contributes to *C. parvum*-associated m6A methylation in infected IEC4.1 cells. We confirmed the downregulation of Alkbh5 and Fto in infected IEC4.1 cells at the RNA level (using real-time PCR, **Figure 2B**) and at the protein level (using Western blot, **Figure 2C**), as well as at the RNA level in isolated intestinal epithelium from neonatal mice of intestinal cryptosporidiosis through oral administration of the parasite (23, 24) (**Figure 2D**), and infected 2D enteroid monolayers from neonatal mouse ileum (**Figure 2E**). Of note, the antibody detected multiple isoforms of Alkbh5 protein. Consistent with results from previous studies (23, 24), upregulation of the inflammatory *Cxcl2* gene was detected in infected IEC4.1 cells as a control (**Figure 2B**). Expression levels of Mettl3 and Mettl14 were with a tendency of decrease in IEC4.1 cells following *C. parvum* infection at 24h, but without statistical significance and this tendency was not observed in other time points following infection (**Figure 2B**). Downregulation of Alkbh5 or Fto was further detected in IEC4.1 cells treated with TNF-α (**Figure S2**) or IFN-α (**Figure S3**).

**Figure 3:**
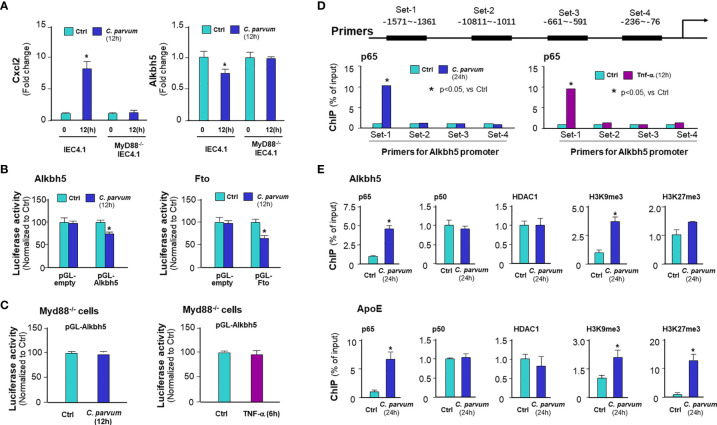
*C. parvum* infection causes downregulation of Alkbh5 and Fto with the involvement of NF-кB signaling activation. **(A)** Downregulation of Alkbh5 in IEC4.1 cells following *C. parvum* infection is MyD88-dependent. Knockout MyD88 in IEC4.1 cells blocked the suppression of Alkbh5 induced by *C. parvum*. Cxcl2 induction in cells in response to infection was also measured for positive control. **(B)** Luciferase activity associated with the promoters of both Alkbh5 and Fto genes in IEC4.1 cells following *C. parvum* infection. Cells were transfected with the generated reporter constructs and then exposed to *C. parvum* infection for 12h, followed by measurement of luciferase activity. Cells transfected with the empty vector were used as control. **(C)** Luciferase activity associated with the promoter of Alkbh5 in MyD88-/- IEC4.1 cells following *C. parvum* infection or TNF-α stimulation. IEC4.1 cells deficient in Myd88 were exposed to *C. parvum* infection (for 12h) or TNF-α stimulation (for 6h). Luciferase activity was measured. **(D)** Recruitment of NF-кB p65 to the Alkbh5 promoter region in IEC4.1 cells following *C. parvum* infection or TNF-α stimulation. Cells were exposed to *C. parvum* infection (for 24 h) or TNF-α stimulation (for 4h), followed by ChIP analysis using anti-p65 and the PCR primer sets as designed. Increased recruitment of p65 was detected in the -1571~-1361 (Set-1) region of the Alkbh5 gene locus in cells following *C. parvum* infection or TNF-α stimulation. **(E)** Recruitment of NF-кB subunits and HDAC1, as well as enrichment of H3K9me3 and H3K27me3, at the Alkbh5 promoter region in intestinal epithelial cells following *C. parvum* infection. Cells were exposed to *C. parvum* infection for 24 h, followed by ChIP analysis using anti-p65, anti-p50, anti-HDAC1, anti-H3K9me3, or anti-H3K27me3 and the PCR primer Set-1. Recruitment of NF-кB subunits and HDAC1, as well as enrichment of H3K9me3 and H3K27me3, at the ApoE promoter region in cells following *C. parvum* infection were also measured as a positive control. Data represent three independent experiments. *p<.05 *vs* the non-infected control.

Given the fact that activation of NF-кB signaling is a common cellular response in intestinal epithelial cells following *C. parvum* infection and upon TNF-α stimulation (25, 36), we asked whether NF-кB signaling is involved in the suppression of Alkbh5 expression in cells following *C. parvum* infection. We exposed IEC4.1 cells deficient in MyD88 (MyD88-knockout, MyD88-KO), one of the key upstream adaptor for pathogen-induced NF-кB activation (25), to *C. parvum* infection and then measured the expression level of Alkbh5. No decrease of Alkbh5 expression level was observed in the MyD88-KO IEC4.1 cells following *C. parvum* infection (**Figure 3A**) or TNF-α stimulation (**Figure S2**). Based on TFSEARCH (http://www.cbrc.jp/research/db/TFSEARCH.html) and MOTIF (http://motif.genome.jp/) database searches (31, 37), putative NF-кB binding sites were identified within the potential promoter region of the Alkbh5 gene. We then cloned the potential promoter regions of the Alkbh5 and Fto genes and inserted the sequences into the pGL-luciferase reporter vector. *C. parvum* infection decreased the luciferase activity in cells transfected with the luciferase construct that encompassed the promoter regions of the *Alkbh5* and *Fto* genes, but not in cells transfected with the empty vector control (**Figure 3B**). Decreased luciferase activity associated with the promoter region of the *Alkbh5* gene induced by *C. parvum* infection was not observed in the MyD88-KO IEC4.1 cells (**Figure 3C**). Moreover, decreased luciferase activity associated with the promoter regions of the *Alkbh5* and *Fto* genes was also detected in IEC4.1 cells following stimulation with TNF-α or IFN-α (**Figure S4**). Since IFN-α stimulation also suppressed Fto expression in IEC4.1 cells, we measured luciferase activity associated with the promoter region of the *Fto* gene in IEC4.1 cells deficient in *Ifnar1* (CRISPR/Cas9 stable knockout cell line, lack of Ifnar1 the receptor subunit for Type I IFN signaling) (38). No significant change in luciferase activity associated with the promoter region of the *Fto* gene were detected in IEC4.1 cells deficient in *Ifnar1* following *C. parvum* infection or IFN-α stimulation (**Figure S4**).

To define how NF-кB signaling suppresses Alkbh5 gene transcription, we performed chromatin immunoprecipitation (ChIP) analysis to measure the occupancy of NF-кB subunits, p65 and p50, to the Alkbh5 gene. An elevated occupancy of p65, but not p50, to the promoter region of Alkbh5 gene locus was detected in IEC4.1 cells following *C. parvum* infection (**Figures 3D, E**) or TNF-α stimulation (**Figure S5**). Previous studies indicate that recruitment of NF-кB subunits to targeted gene promoters may promote occupancy of suppressive histone deacetylase 1 (HDAC1) to suppress gene transcription (39, 40). However, no increase of HDAC1 occupancy was detected in the promoter region of Alkbh5 gene in infected IEC4.1 cells (**Figure 3E**) or cells following TNF-α stimulation (**Figure S5**). An enrichment of H3K9me3, but not H3K27me3, to the promoter region of Alkbh5 gene locus was observed in IEC4.1 cells following *C. parvum* infection (**Figure 3E**) or TNF-α stimulation (**Figure S5**). Consistent with results from previous studies (41, 42), the enrichment of p65, H3K9me3, and H3K27me3 to the promoter region of ApoE gene locus, an NF-кB-associated downregulating gene, was detected in infected IEC4.1 cells as a control (**Figure 3E**). These data suggest that NF-кB signaling may count for the suppression of Alkbh5 in cells following *C. parvum* infection or TNF-α stimulation.

### m^6^A Methylation Statuses Influence Intestinal Epithelial Innate Defense Against *C. parvum* Infection

**Figure 4:**
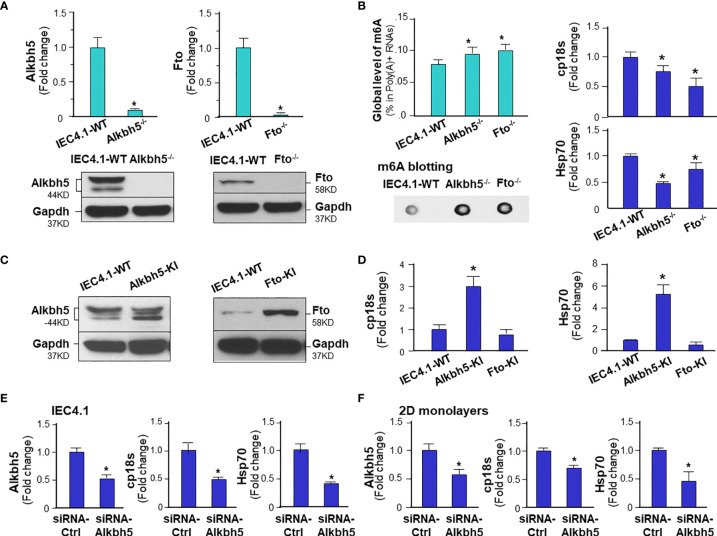
m^6^A methylation modulates intestinal epithelial innate defense against *C. parvum* infection. **(A)** Knockdown of Alkbh5 or Fto in IEC4.1 cells. Cells were transfected with the CRISPR/Cas9 KO(h) for Alkbh5 or Fto and the HDR plasmids. Stable transfected cells were cloned and confirmed by real-time PCR and Western blot analysis. Gapdh was also blotted for control. **(B)** Knockdown of Alkbh5 or Fto in IEC4.1 cells decreased the infection burden of *C. parvum* infection. Knockdown of Alkbh5 or Fto increased m^6^A RNA methylation in IEC4.1 cells, as measured by m6A RNA methylation quantitation assay and dot blot. IEC4.1 cells and cells deficient with Alkbh5 or Fto were then exposed to *C. parvum* infection for 24 h. IEC4.1 cells transfected with the empty vector, marked as IEC4.1-WT (wild type) were used as the control. Infection burden of *C. parvum* was quantified by measuring parasite cpHsp70 or cp18s using real-time PCR. **(C)** Knock-in of Alkbh5 or Fto in IEC4.1 cells. Cells were transfected with the CRISPR/Cas9 KO(h) for active Alkbh5 (Alkbh5-KI) or Fto (Fto-KI) vectors. Stable transfected cells were cloned and confirmed by Western blot analysis. **(D)** Overexpression of Alkbh5, but not Fto, increased the infection burden of *C. parvum* in IEC4.1 cells. Cells stably expressing Alkbh5 or Fto were exposed to *C. parvum* infection for 24 h and infection burden of *C. parvum* was quantified. **(E)** Knockdown of Alkbh5 *via* siRNA decreased the infection burden of *C. parvum* in IEC4.1 cells. Cells were treated with the siRNA to Alkbh5 for 24 h and exposed to *C. parvum* infection for additional 24 h. Cells treated with the non-specific scrambled siRNA were used as the control and infection burden of *C. parvum* was quantified. **(F)** Knockdown of Alkbh5 *via* siRNA decreased the infection burden of *C. parvum* in 2D intestinal epithelial monolayers. Monolayers were cultured and treated with the siRNA to Alkbh5 for 24 h and exposed to *C. parvum* infection for additional 24 h. Cells treated with the non-specific scrambled siRNA were used as the control and infection burden of *C. parvum* was quantified. Data represent three independent experiments. *p<.05 *vs* cells transfected with the empty-vector control (as IEC4.1-WT in **A, B, D**) or cells treated with the control-siRNA (in **E, F**).

Given the key role of the NF-кB signal pathway in innate antimicrobial defense (43), we reasoned if m^6^A methylation can modulate intestinal epithelial cell defense against *C. parvum* infection. To address this possibility, we took the CRISPR/Cas9 knock-out approach to establish stable IEC4.1 cells deficient in *Alkbh5* or *Fto*. Knock-out of *Alkbh5* and *Fto* in IEC4.1 cells were confirmed by real-time PCR and Western blot (**Figure 4A**). Accordingly, knockout of *Alkhb5* or *Fto* caused a significant increase of global m^6^A mRNA methylation in IEC4.1 cells (**Figure 4B**). Cells were then exposed to *C. parvum* infection for measurement of attachment/invasion (after incubation for 4h) and host anti-parasite defense (so called infection burden, after incubation for 24 or 48h), as previously reported (44). A decreased infection burden was detected in IEC 4.1 cells deficient in *Alkbh5* or *Fto* (**Figure 4B**). We then took the CRISPR/Cas9 knock-in approach to establish stable IEC4.1 cells to overexpress Alkhb5 or Fto (**Figure 4C**). Cells expressing Alkbh5 showed an increase of infection burden (**Figure 4D**). Intriguingly, an increase of infection burden was not detected in cells constitutively expressing Fto (**Figure 4D**). No obvious difference in the attachment/invasion of *C. parvum* was observed in cells deficient in *Alkbh5* or *Fto* and in cells overexpressing Alkbh5 or Fto, compared with that in the control IEC4.1 cells (**Figure S6**). Moreover, siRNAs to knockdown *Alkbh5* also decreased the burden of *C. parvum* infection in IEC4.1 cells (**Figure 4E**) and in 2D intestinal monolayers derived from neonatal mice (**Figure 4F**).

### Alterations in the Topology of Host m^6^A mRNA Methylome in Intestinal Epithelial Cells Following *C. parvum* Infection

**Figure 5:**
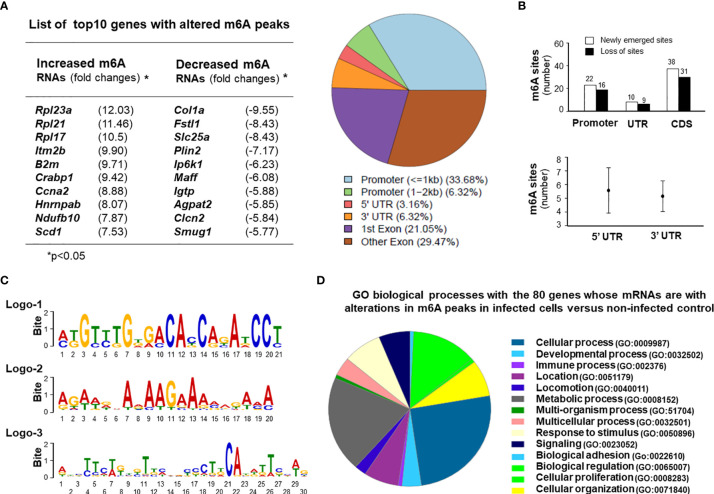
Alterations in the topology of host m^6^A mRNA methylome in intestinal epithelial cells following *C. parvum* infection. **(A)** *C*. *parvum* infection caused significant alterations in m^6^A levels in the 118 regions of 80 corresponding genes in the transcriptome in IEC4.1 cells. Cells were exposed to *C. parvum* infection for 24h. Total mRNAs were collected and processed for m^6^A-RIP-Seq analysis. Top 10 genes with altered m^6^A modifications (either with an increased m^6^A peaks or a decreased m^6^A peaks) in infected cells are listed. These regions at the promoters, untranslated regions (UTRs) and coding sequence (CDS) regions with significant increase or decrease of m^6^A peaks from the infected cells are shown. **(B)** Sites of altered m^6^A methylation in the promoters, UTRs, and CDS regions in cells following *C. parvum* infection. **(C)** The motifs with altered m^6^A modifications in IEC4.1 cells following infection. Three logos were identified and listed. **(D)** Gene ontology (GO) analysis of the genes with changed m^6^A peaks in cells following *C. parvum* infection.

We next examined the topology of host m^6^A mRNA methylome in IEC4.1 cells following *C. parvum* infection by performing methylated RNA immunoprecipitation sequencing (MeRIP-seq) experiments. For this, IEC4.1 cells were exposed to *C. parvum* infection for 24h. Total mRNA transcripts were isolated and processed for m^6^A sequencing (m^6^A-seq) experiments, as previously reported (4, 45). We first examined the abundance and distribution of m^6^A peaks on host mRNA transcripts from uninfected and *C. parvum*-infected cells. Metagene analysis showed that *C. parvum* infection caused significant alterations in m^6^A peaks in the 118 regions of 80 corresponding genes in the transcriptome (**Figure 5A** and **Table S1**). The top ten genes with a significant alteration in their m^6^A peaks are shown in **Figure 5A** and the complete 118 regions and corresponding genes are listed in **Table S1**. Of these regions, the majority are at the promoter regions (<1kb 33.68% and 1-2kb 6.32%) and the coding sequence (CDS) regions (1st exon 21.05% and other exon 29.47%) of the target genes (**Figure 5A**). The other peaks are at the 3’UTR (6.32%) and 5’UTR (3.16%) regions (**Figure 5A**). Newly emerged m^6^A methylation sites were detected in 22 promoter regions, 10 UTR regions and 38 CDS regions (**Figure 5B**). Loss of exiting m^6^A methylation sites was detected in 16 promoter regions, 9 UTR regions and 31 CDS regions (**Figure 5B** and **Table S2**). It appears that no significant difference of alterations in m^6^A peaks was observed between the 5’UTR and 3’UTR regions (**Figure 5B**). We also performed a motif analysis of the newly emerged and lost m^6^A peaks in cells following *C. parvum* infection. It revealed three top motifs (**Figure 5C**). The distribution of motifs covered over the promoters, UTRs and CDS regions, with varies in different genes (**Figure S7**). All sequence data were described in accordance with MIAME guidelines and deposited at NCBI database (with the NCBI accession numbers: SRR14029773 - SRR14029784).

Gene ontology analysis of the genes with changed m^6^A peaks identified a broad range of gene categories among the most enriched pathways in both the newly gained and lost m^6^A methylation sites, including immune-related genes, genes for RNA splicing and translation, mitochondrion functions, and cell proliferation (**Figure 5D** and **Table S1**). These immune-related genes include *Igtp, Irgm2, Cx3cl1, Crabp1, Iqgap1*, and *Jmid8*. Genes involving with RNA translation and splicing include *Rpl21, Rpl23q, Rpl17, Rpl12, Cep85, Hnrnpab, Rbm8a*, and *Sf3b1*. Genes associated with mitochondrion functions include *Ndufb10, Idh3b, Wdr90, Bcap31*, and *Uqcrb*. Cell proliferation-related genes include *Sf3b1, Fosl2, Ccna2, Plcd3, Fanca, Flna, Btc*, and *Sipa1* (**Figure 5D** and **Table S1**).

### mRNA Expression Profile and Its Association With m^6^A Peaks in Intestinal Epithelial Cells Following *C. parvum* Infection

**Figure 6:**
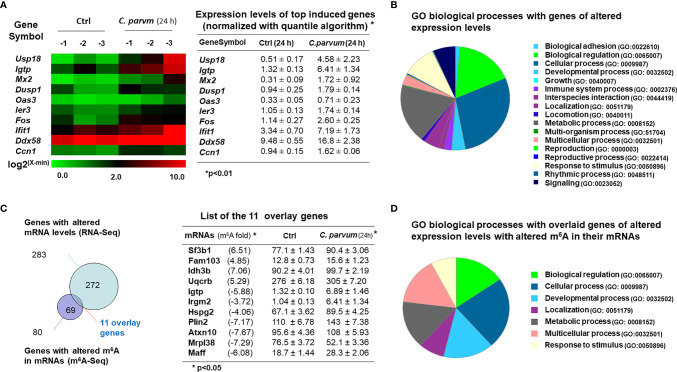
mRNA expression profile and its association with m^6^A levels in intestinal epithelial cells following *C. parvum* infection. **(A)** Heatmaps representing upregulation of the top 10 genes in IEC4.1 cells following *C. parvum* infection. IEC4.1 cells were exposed to *C. parvum* infection for 24 h followed by genome-wide RNA-Seq analysis. **(B)** Gene ontology (GO) analysis of genes whose expression levels were significant altered in IEC4.1 cells revealed by RNA-Seq analysis. **(C)** Comparison of genes whose expression levels were altered and these genes whose RNAs were with altered m^6^A levels in infected IEC4.1 cells. Only a small portion of the genes was overlaid. **(D)** Gene ontology (GO) analysis of the overlaid genes of altered expression levels and with altered m^6^A modifications.

Of these mRNAs isolated from uninfected and infected IEC4.1 cells and processed for m^6^A-Seq analysis as described above, we also took a portion of the mRNA collections for whole genome transcriptome (RNA-Seq) analysis. Consistent with results from previous studies, we detected many genes that were upregulated or downregulated in cells following infection. The top 10 induced genes are listed in **Figure 6A** and a full list of upregulated and downregulated genes is provided in **Table S2**. These upregulated genes include immune-related genes (e.g., *Mx2, Igtp, Iift1, Ddx58*, and *Cxcl1*), stress-responsive genes (*Usp18, Oas3, Ier3, Cox7a1*, and *Uba7*), and metabolism-related genes (*Dusp1, Fos, Beu1, Gbp2, Zfp36, Wnt4*, and *Dtx3l*) (**Figure 6B** and **Table S2**). All sequence data were described in accordance with MIAME guidelines and deposited at NCBI database (with the NCBI accession numbers: SRR14163429 - SRR14163434).

Interestingly, comparison of genes whose expression levels were altered and genes with altered m^6^A methylation in infected IEC4.1 cells revealed that only a small portion of the genes was overlaid (**Figure 6C**). This represents 13.75% of the genes with altered m^6^A levels and 3.89% of the genes those expression levels are either upregulated or downregulated in cells following *C. parvum* infection for 24h (**Figure 6C**). The majority of genes with increased or decreased m^6^A levels did not show a significant change in their expression levels in cells following *C. parvum* infection. Representative overlay genes include *Irgm2, Igtp, Sf3b1, Rbm8a*, and *Idh3b* (**Figure 6D**). There was no obvious correlation between their expression levels and the m^6^A site locations, such as m^6^A in the promoters, UTRs or CDS regions (data not shown). Moreover, gene ontology analysis of these 11 genes with altered expression levels and altered m^6^A methylation revealed board biological processes, including cell adhesion, metabolic and immune processes (**Figure 6D**).

### Expression Levels of Two Immunity-Related GTPase Genes, Irgm2 and Igtp, Were Increased With a Decreased m^6^A mRNA Methylation in IEC4.1 Cells Following *C. parvum* Infection

**Figure 7:**
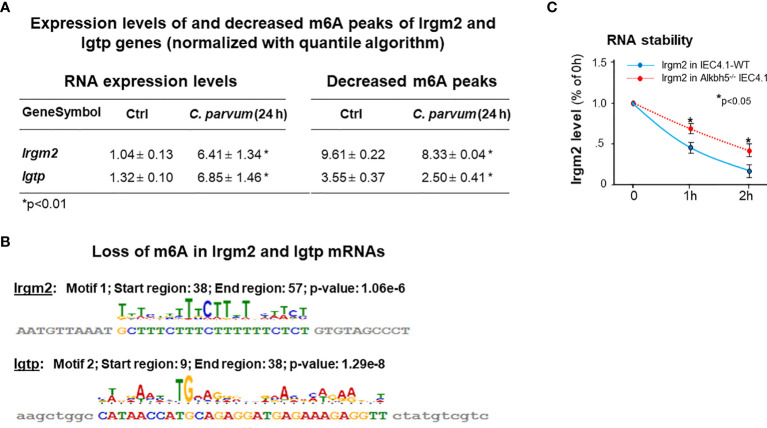
Expression levels of Irgm2 and Igtp are increased and with a decreased m^6^A methylation in IEC4.1 cells following *C. parvum* infection. **(A)** Increased expression levels of both Irgm2 and Igtp genes with a decreased m^6^A peaks in their mRNAs in IEC4.1 cells following *C. parvum* infection. RNA levels of Irgm2 and Igtp and their m^6^A levels were assessed by RNA-Seq and m^6^A-RIP-Seq, respectively. **(B)** Decrease in their m^6^A levels in cells following *C. parvum* infection occurred in the promoters/UTRs/CDS regions. **(C)** Increased RNA stability of Irgm2 mRNA in Alkbh5-/- IEC4.1 cells versus that in IEC4.1 cells. Data represent three independent experiments. *p<.05 *vs* the non-infected control or cells transfected with the empty-vector control (as IEC4.1-WT in **C**).

Interestingly, we found out that expression levels of Irgm2 and Igtp (also called as Irgm3 in mice) were increased in infected cells. Given the fact that suppression of m^6^A methylation in IEC4.1 cells results in an increase of *C. parvum* burden, coupled with the important role of Irgm2 and Igtp in innate epithelial immunity (46, 47), we looked more details about m^6^A methylation for the genes of Irgm2 and Igtp in infected cells. We found out that both Irgm2 (NM_019440) and Igtp (NM_018738) mRNAs showed a decrease in their m^6^A methylation in cells following *C. parvum* infection, as revealed by m^6^A-seq (**Figure 7A**). Relevant motif and distribution of decreased m^6^A peaks in Irgm2 and Igtp mRNAs are shown in **Figure 7B**. Moreover, increased stability of Irgm2 mRNA was observed in Alkbh5-/- IEC4.1 cells, compared with that in IEC4.1 cells (**Figure 7C**).

### m^6^A Methylation-Mediated Intestinal Epithelial Anti-*C. parvum* Defense in Human Intestinal Epithelium

**Figure 8:**
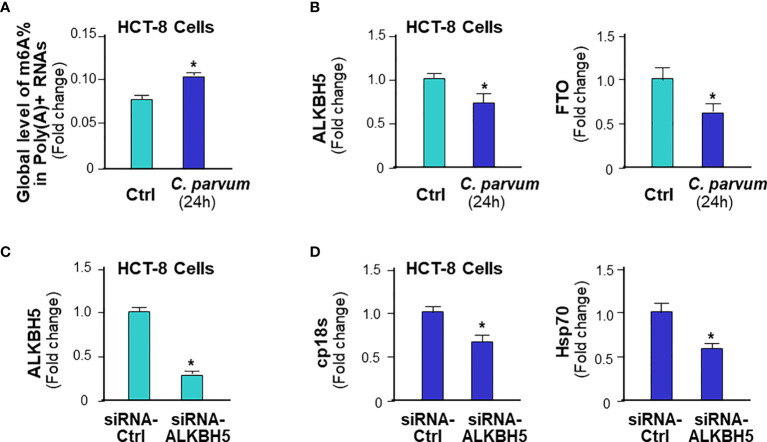
m^6^A methylation-mediated intestinal epithelial anti-*C. parvum* defense in human intestinal epithelium. **(A)** Increase of global m^6^A RNA methylation in HCT-8 cells following *C. parvum* infection. Cells were exposed to *C. parvum* infection for 24h and m^6^A RNA methylation was measured by m^6^A RNA methylation quantitation assay. **(B)** Decrease of ALKBH5 and FTO expression levels in HCT-8 cells following *C. parvum* infection. Cells were exposed to *C. parvum* infection for 24h and expression levels of ALKBH5 and FTO was measured by real-time PCR. **(C)** Knockdown of ALKBH5 *via* siRNA in HCT-8 cells. Cells were treated with the siRNA to ALKBH5 for 24h and knockdown of ALKBH5 was confirmed by real-time PCR. Cells transfected with non-specific control siRNA were used as the control. **(D)** Knockdown ALKBH5 in HCT-8 cells decreased *C. parvum* infection burden. HCT-8 cells were first treated with the siRNA-ALKBH5 and then exposed to *C. parvum* infection for 24h. Infection burden of *C. parvum* was quantified by measuring parasite cpHsp70 or cp18s using real-time PCR. Data represent three independent experiments. *p<.05 *vs* the non-infected control (in **A–D**).

Using an *in vitro* infection model employing human intestinal epithelial HCT-8 cells (48), we further tested the role of ALKBH5-mediated m^6^A mRNA methylation in human intestinal epithelial anti-*C. parvum* defense. Increase of global m^6^A RNA methylation statute was detected in HCT-8 cells following infection (**Figure 8A**). Decrease of ALKBH5 and FTO expression levels was detected in HCT-8 cells following *C. parvum* infection (**Figure 8B**). The impact of knockdown ALKBH5 on *C. parvum* infection burden was further confirmed in HCT-8 cells. We took the siRNA approach to knockdown ALKBH5 in HCT-8 cells (**Figure 8C**). When HCT-8 cells were treated with the siRNA-ALKBH5 and then exposed to *C. parvum* infection for 24h, a significant decrease of infection burden was observed (**Figure 8D**).

## Discussion

In this study, we present data demonstrating significant alterations in the topology of host m^6^A mRNA methylome in intestinal epithelial cells in response to *C. parvum* infection. *C. parvum* infection promotes a global increase of m^6^A mRNA methylations in infected host cells through downregulation of Alkbh5 with the involvement of NF-кB signaling. Whereas global m^6^A methylation in infected host cells promotes epithelial anti-*C. parvum* defense, most mRNAs with increased or decreased m^6^A methylation levels do not show a significant change in their expression levels in infected cells. However, elevated expression levels of specific immune-related genes, such as *Irgm2* and *Igtp*, are correlated with a decreased m^6^A mRNA methylation in infected cells. Our data support that intestinal epithelial cells display significant alterations in the topology of their m^6^A mRNA methylome in response to *C. parvum* infection with the involvement of activation of the NF-кB signaling pathway, which may contribute to fine regulation of epithelial anti-*C. parvum* defense.

m^6^A dynamics are finely controlled by various methyltransferases (or writers) and demethylases (or erasers) (2, 3, 7). Key methyltransferases are METTL3 and METTL14 and important demethylases include FTO, ALKBH3, and ALKBH5 (2, 3, 7). Previous studies have demonstrated that mammalian cells have developed strategies to modulate cellular m^6^A RNA methylation statutes in response to heat shock (49) or viral infection (11, 50). However, little is known about the molecular mechanisms of how extracellular stimuli may activate intracellular signals to modulate cellular m^6^A RNA methylation. Our data indicate that downregulation of Alkbh5 and Fto may account for the elevated m^6^A methylation in murine intestinal epithelial cells in response to *C. parvum* infection. Interestingly, downregulation of the *Alkbh5* gene involves the activation of the NF-кB pathway in infected cells. Recruitment of NF-кB p65 subunit and enrichment of suppressive marker H3K9me3 to the promoter region of the *Alkbh5* gene maybe associated with its downregulation. Activation of TLR/MYD88/NF-кB pathway has previously been demonstrated in epithelial cells following *C. parvum* infection (25). Indeed, knockdown of MyD88 blocked the downregulation of the *Alkbh5* gene in infected cells. Moreover, since the TLR/MyD88/NF-кB pathway can be activated following infection by many pathogens, it is plausible that regulation of m^6^A methylation through activation of TLR/MyD88/NF-кB signaling may be a general cellular response to microbial infection. Similarly, downregulation of Alkbh5 was previously reported in epithelial cells in response to infection by *Streptococcus suis* (51), *H1N1 influenza* virus (52), and *Chlamydia pneumoniae* (53). An increase of global m^6^A methylation was also found in epithelial cells following infection by SARS-CoV-2 virus (54) or Kaposi’s sarcoma-associated herpesvirus (55), and in immune cells by various pathogens (56). Moreover, we observed both elevated m^6^A methylation and lost m^6^A peaks at the UTR and CDS regions of target genes. The motif usage changes to these regions seem to occur on the overall level in cells following *C. parvum* infection. This suggests that not only the erasers but also the writers, including Mettl3 and Mettl14, may be involved in the regulation of m^6^A methylation in cells following *C. parvum* infection.

RNA m^6^A methylation regulates RNA splicing, translocation, stability, and translation into protein (3–6). These genes with changed m^6^A peaks identified in *C. parvum*-infected cells cover a broad range of gene categories among the most enriched pathways in both the newly gained and lost m^6^A methylation, including immune-related genes, genes for RNA splicing and translation, mitochondrion functions, and cell proliferation (57–61). Activation of innate epithelial defense and dysfunction of mitochondrion and cell proliferation have previously demonstrated in intestinal epithelial cells following *C. parvum* infection (62, 63). Therefore, *C. parvum* infection might affect gene translation, alternative splicing, and mRNA stability, as a consequence of differential deposition of m^6^A methylation. Particularly, the effects of m^6^A methylations on RNA stability would directly affect the expression levels of target RNAs (4). Interestingly, comparison of genes whose expression levels were altered and genes with an altered m^6^A levels in infected host cells revealed that only a small portion of the genes was overlaid. The majority genes with increased or decreased m^6^A levels did not show a significant change in their expression levels in cells following *C. parvum* infection. This clearly indicates that modulation of RNA stability may be one of the many mechanisms that m^6^A methylation can regulate cellular function. Other mechanisms may involve RNA splicing and translation associated with m^6^A methylation of target mRNAs.

Another key finding of this study is the observation that elevated m^6^A methylation promotes intestinal epithelial innate defense against *C. parvum* infection both in mice and in humans. Manipulation of Alkbh5 expression levels through the CASPR/Cas9 knock-out and knock-in approach caused reciprocal alterations in global m^6^A mRNA methylation in host cells, and consequently, infection dynamics of *C. parvum in vitro*. It is unclear why an increase of infection burden was not detected in cells constitutively expressing Fto, whereas a decreased infection burden was detected in cells deficient in Fto. Since the parasite attachment/invasion of host cells appears not affected by the genomic manipulation of Alkbh5 or Fto, it is plausible to speculate that m^6^A methylation may regulate innate intestinal epithelial anti-*C. parvum* defense. In this study, we did not include analysis of m6A methylation in the *C. parvum* RNA transcriptome, which may also undergo specific m^6^A methylations and thus, modulates host-parasite interactions. Multiple m^6^A methylation sites have been identified in the viral RNA genome and transcripts of DNA viruses in recent years (64). Several families in nonsegmented negative-sense RNA viruses acquire m^6^A in viral RNA as a common strategy to evade host innate immunity (65).

We identified Irgm2 and Igtp, two immunity-related GTPase genes whose expression levels were induced with a decreased RNA m^6^A methylation in *C. parvum*-infected murine intestinal epithelial cells. Both Irgm2 and Igtp proteins are members of the immunity-related GTPases, a family of large, interferon-inducible GTPases implicated in resistance against a wide variety of intracellular pathogens, including *Toxoplasma gondii, Leishmania major, Trypanosoma cruzi, Chlamydia trachomatis*, *C. psittaci, Mycobacterium tuberculosis, M. avium, Salmonella typhimurium, Listeria monocytogenes*, and *Legionella pneumophila* (66–71). Igtp/Irgm3 knockout mice are significantly more susceptible to *T. gondii* infection than their wild-type counterparts (67). Whereas little is known regarding potential functions of this family in the infection of extracellular pathogens, GTPase family members seem to have essential and pathogen-specific roles in resistance to infections (72). Irgm2 may play a role in the innate immune response by regulating autophagy formation in response to intracellular pathogens (70). In addition, increasing evidence supports that GTPase family represents a new IFN-γ-dependent, nitric oxide synthase 2-independent pathway in the control of pathogen infection (68, 73). Our data suggest that induction of Irgm2 and Igtp may also be associated with their m^6^A methylation in intestinal epithelial cells in response to *C. parvum* infection. Given the key role of the TLR/MyD88/NF-кB signal in epithelial innate antimicrobial defense (43), coupled with the modulation of m^6^A methylation through TLR/MyD88/NF-кB signaling in *C. parvum*-infected cells, our data implicate a new mechanism by which TLR/MyD88/NF-кB signaling coordinates intestinal epithelial antimicrobial defense. In addition, both Irgm2 and Igtp are critical modulators for IFN signaling (70). Their induction in intestinal epithelial cells following infection may provide a new cross-link for the network between m^6^A RNA methylation, TLR/MyD88/NF-кB and IFN signaling to modulate intestinal epithelial against *C. parvum*, relevant to fine regulation of epithelial antimicrobial defense in general.

## Data Availability Statement

The datasets presented in this study can be found in online repositories. The names of the repository/repositories and accession number(s) can be found in the article/**Supplementary Material**.

## Ethics Statement

The animal study was reviewed and approved by Creighton University IACUC Committee.

## Author Contributions

ZX, GL, and X-MC designed experiments and wrote the manuscript. ZX, JX, WH, SD, A-YG performed experiments. ZX, JX, EL, JS-S, GM, GL, and X-MC performed data analysis. A-YG and X-MC directed and supervised the study. All authors contributed to the article and approved the submitted version.

## Funding

This work was supported by funding from the National Institutes of Health (AI116323, AI136877, AI141325, and AI156370 to X-MC). The project described was also supported by Grant Number G20RR024001 from the National Center for Research Resources. The content is solely the responsibility of the authors and does not necessarily represent the official views of the National Center for Research Resources or the National Institutes of Health.

## Conflict of Interest

The authors declare that the research was conducted in the absence of any commercial or financial relationships that could be construed as a potential conflict of interest.

## Supplementary Material

Supplementary Table 1 List of 118 regions with altered m^6^A peaks in the corresponding 80 genes in infected cells revealed by m^6^A-RIP-Seq analysis.
