# Supplementary figures and images for: m6A mRNA Methylation Regulates Epithelial Innate Antimicrobial Defense Against Cryptosporidial Infection
Source: Front Immunol. 2021 Jul 6;12:705232. doi: 10.3389/fimmu.2021.705232 (PMC8291979; doi:10.3389/fimmu.2021.705232)

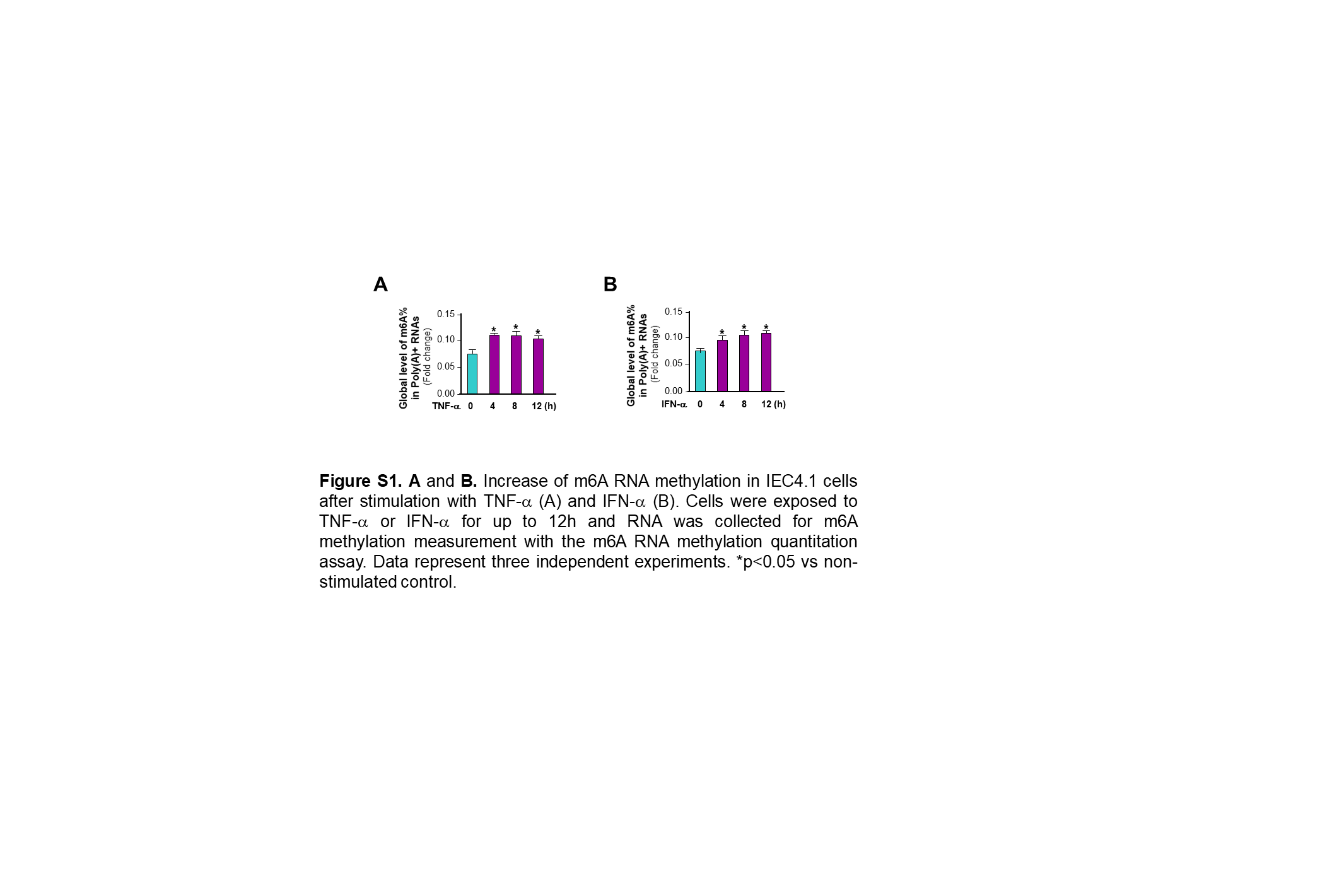

Supplement: Supplementary file 5 [file Image_1.tif]

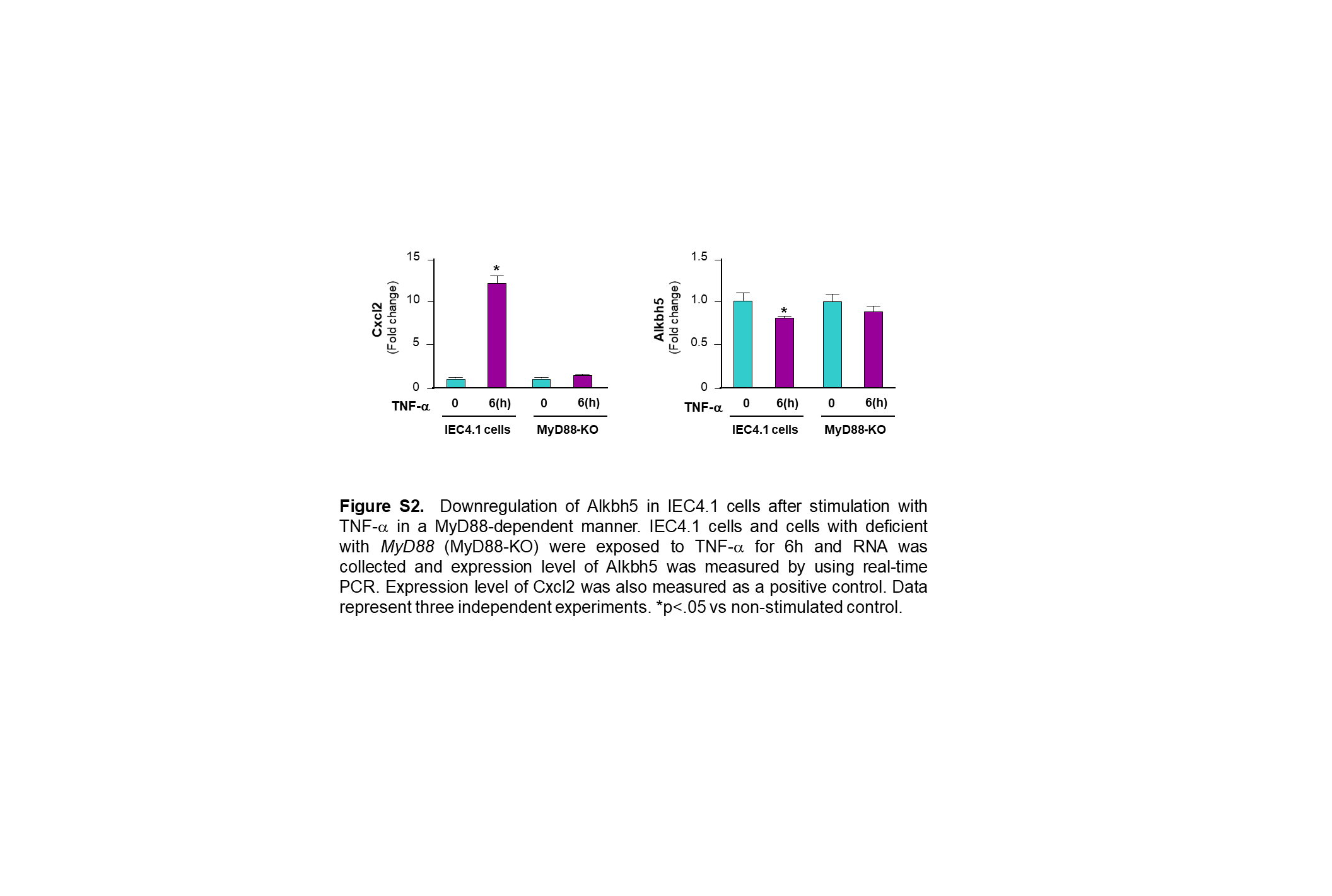

Supplement: Supplementary file 6 [file Image_2.tif]

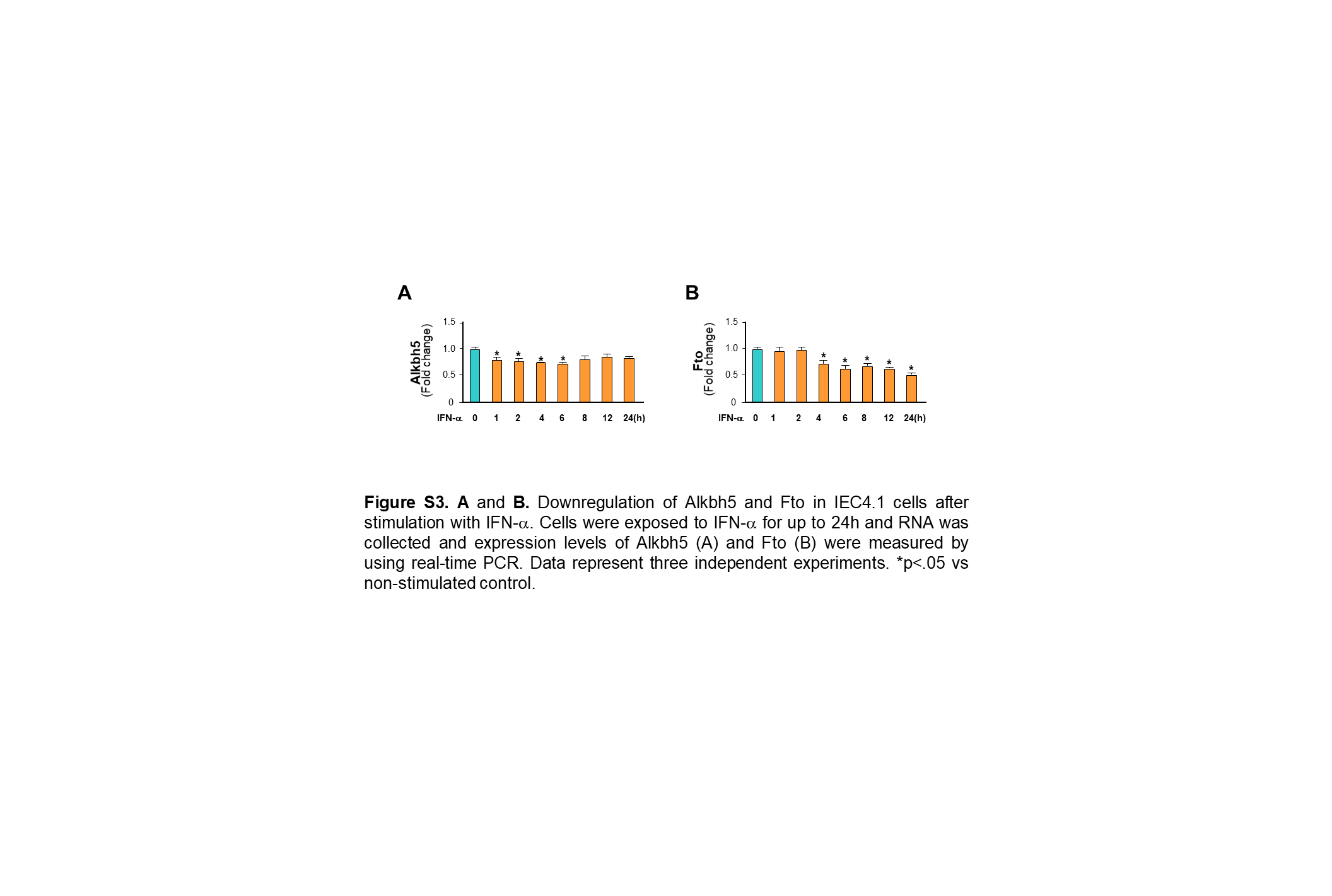

Supplement: Supplementary file 7 [file Image_3.tif]

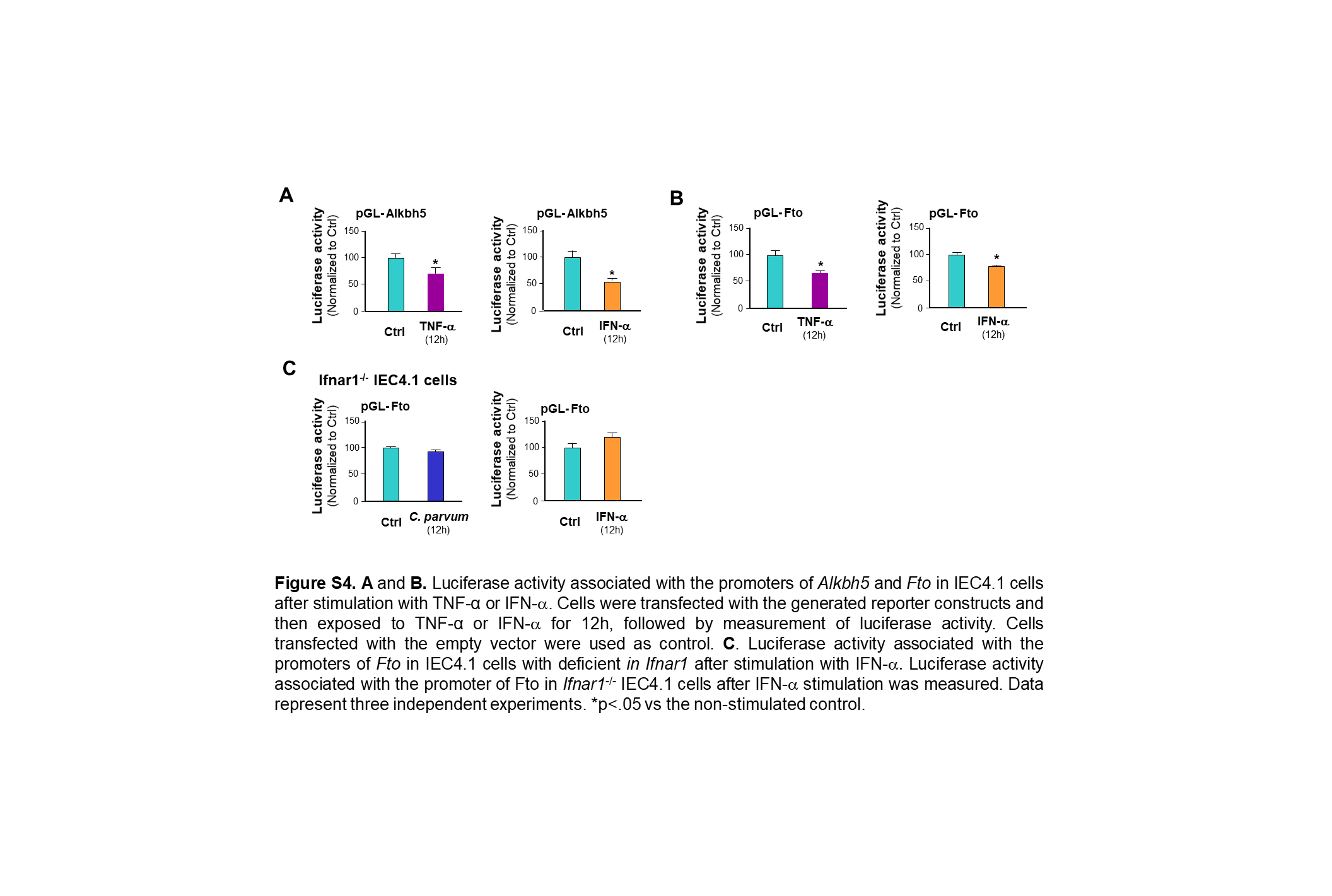

Supplement: Supplementary file 8 [file Image_4.tif]

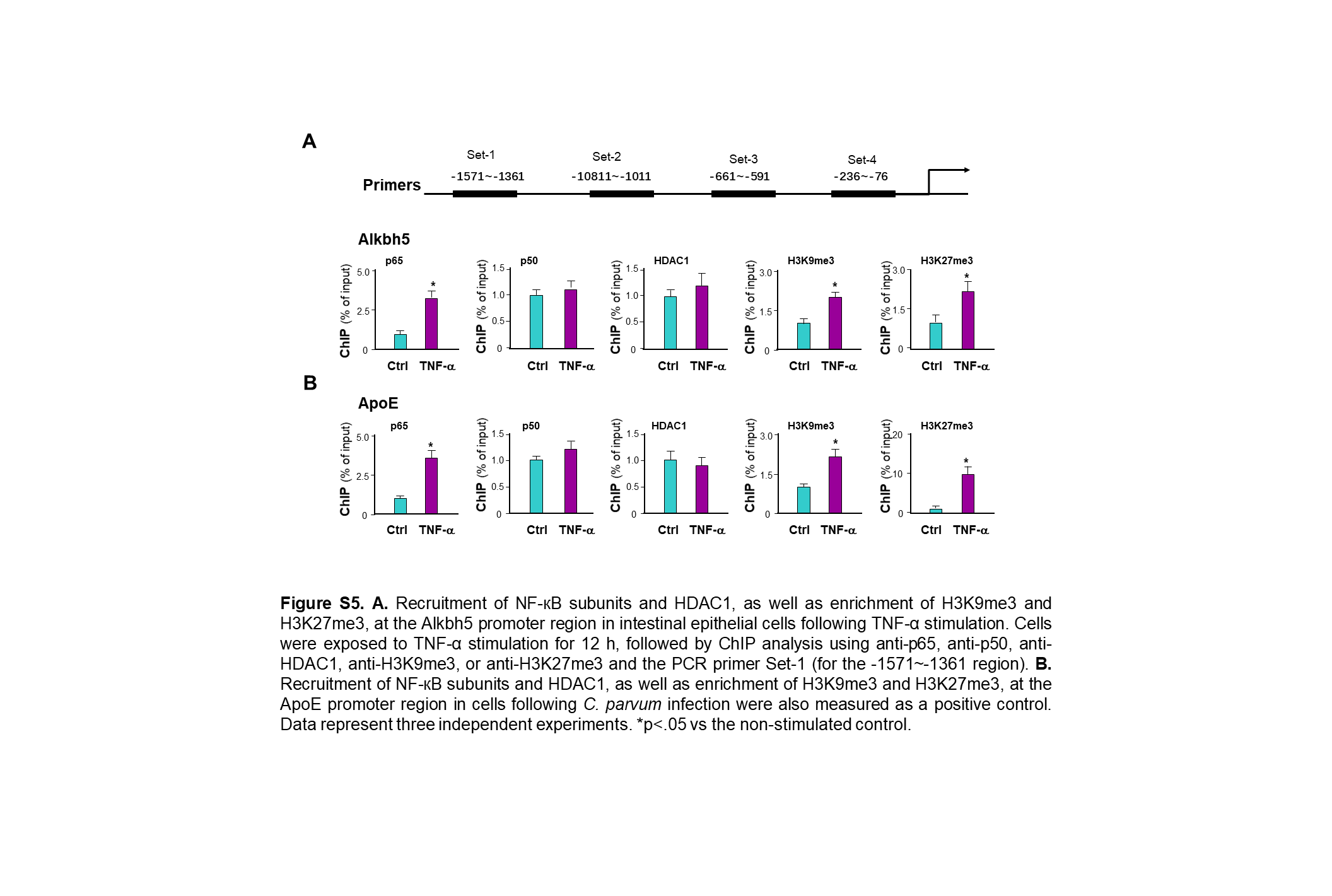

Supplement: Supplementary file 9 [file Image_5.tif]

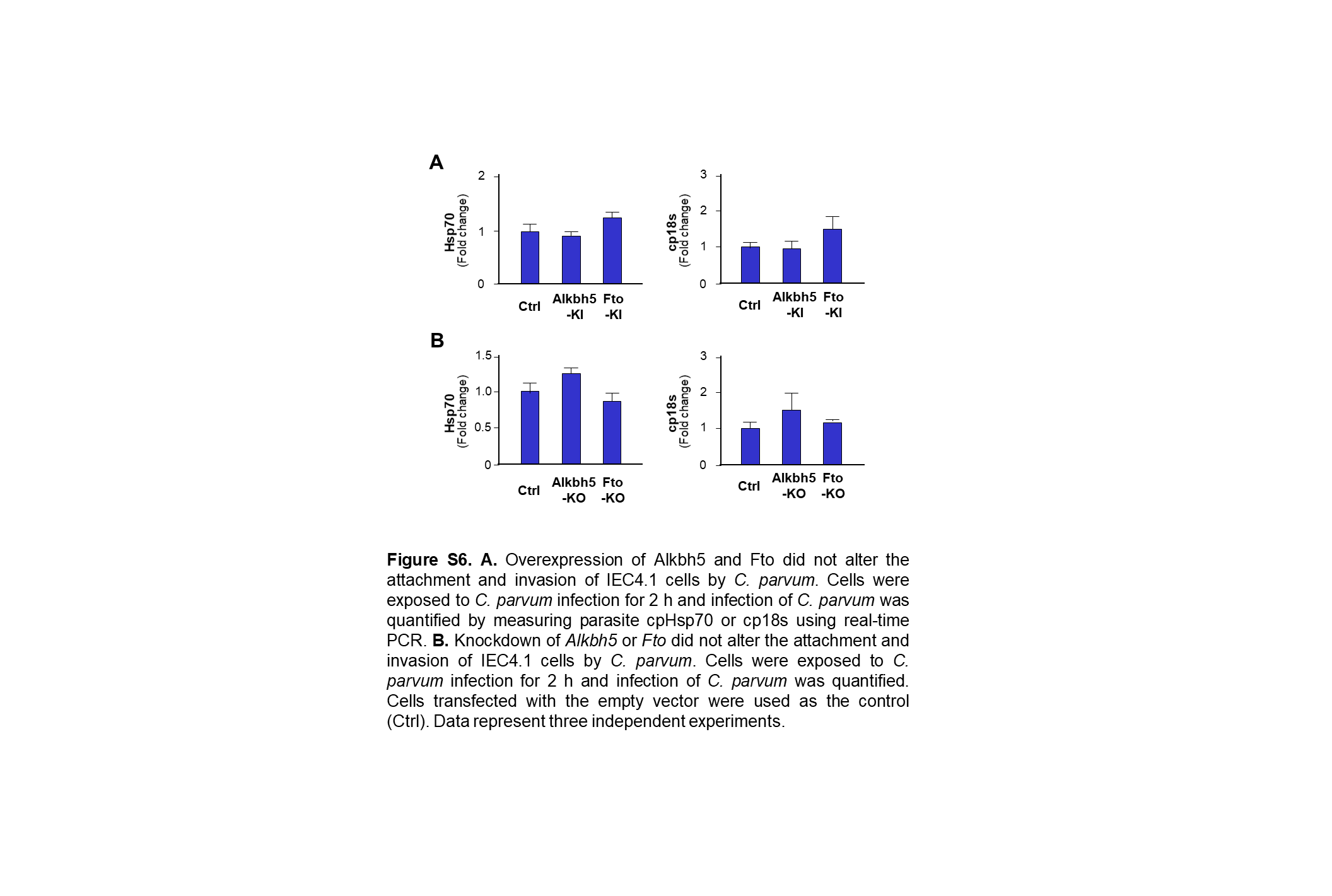

Supplement: Supplementary file 10 [file Image_6.tif]

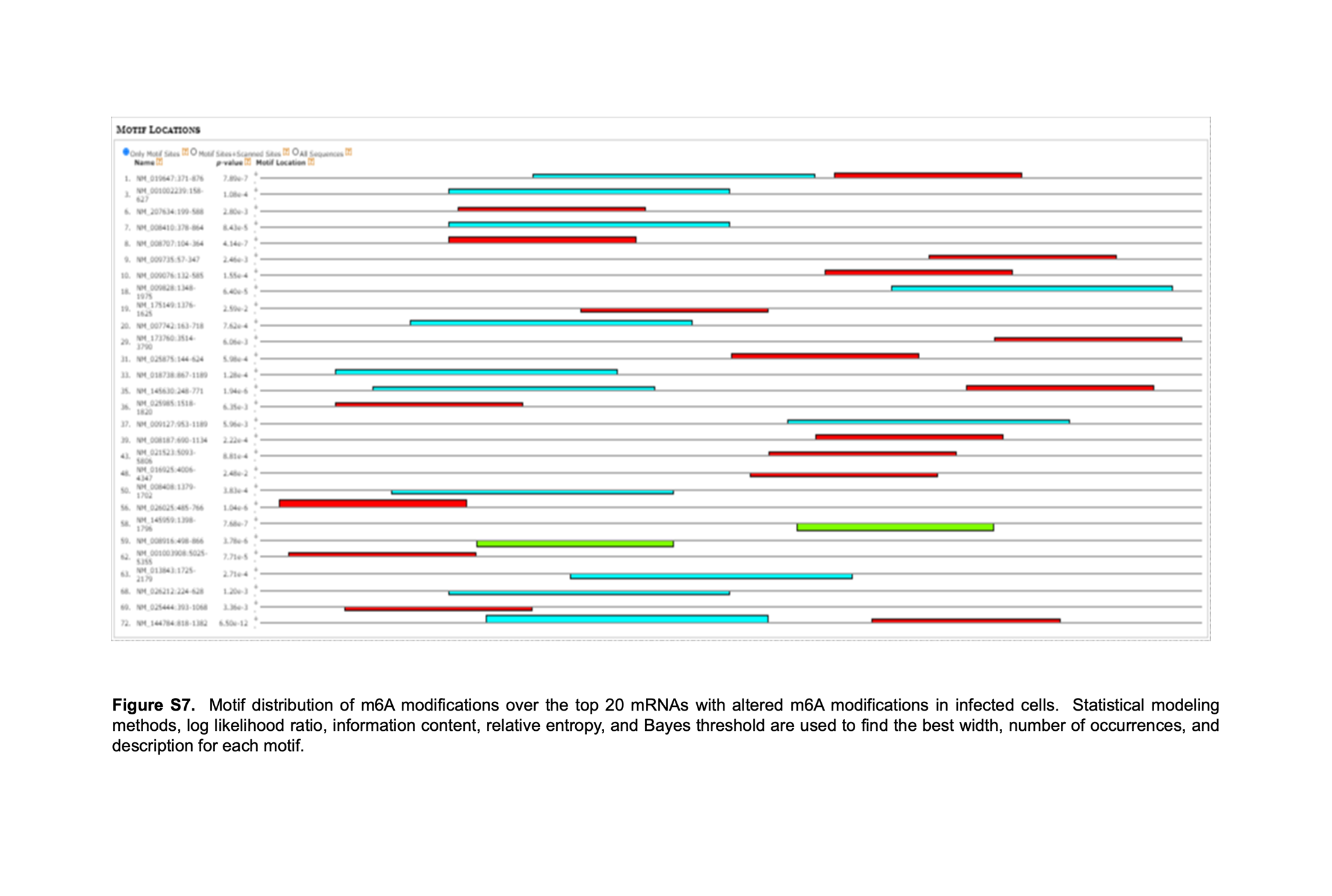

Supplement: Supplementary file 11 [file Image_7.tif]
