# Supplementary material for: m6A mRNA Methylation Regulates Epithelial Innate Antimicrobial Defense Against Cryptosporidial Infection
Source: Front Immunol. 2021 Jul 6;12:705232. doi: 10.3389/fimmu.2021.705232 (PMC8291979; doi:10.3389/fimmu.2021.705232)
Supplement: Supplementary file 12 [file DataSheet_1.docx]

**Supplemental Experimental Procedures**

**RNA Stability**

RNA stability assay was performed by real-time PCR as previously reported (Subramaniam et al., 2008; Zhou et al., 2009). Briefly, cells were exposed to *C. parvum* infection for 24h and transcription was then blocked using actinomycin D (10 µg/ml, Sigma). RNAs were isolated at various time points after actinomycin D treatment. Real-time PCR was then performed using 500 ng of template cDNA for each mRNA gene of interest. Each sample was run in triplicate. The relative abundance of each mRNA was calculated using the ΔΔCt method and normalized to Gapdh. The relative amount of mRNA at 0h following actinomycin D treatment was arbitrarily set to 1. Curve fittings of the resultant data were performed using Microsoft Excel and the half-lives of the RNAs calculated.

**ChIP Analysis**

The formaldehyde crosslinking ChIP was performed as described (Hu et al., 2016; Tong et al., 2016). Briefly, cells in culture were first treated with trypsin, washed once with culture medium containing 10% FBS, washed twice with 10 ml PBS, and resuspended in 10 ml of PBS. Formaldehyde (37% stock solution) was then added to a final concentration of 1 % (v/v) and incubated at 37 ˚C for 10 min with slow mixing. Crosslinking reactions were quenched by the addition of glycine (pH7.0) to a final concentration of 0.25 M followed by incubation at room temperature for 5 min. The cells then harvested by centrifugation using a clinical centrifuge at 3000 rpm (237g) for 4 min followed by two washes with ice-cold PBS. Cell pellets were resuspended in 500 µl of WCE buffer (20 mM HEPES, pH 7.4, 0.2 M NaCl, 0.5% Triton X-100, 10% glycerol, 1 mM EDTA, 1 mM EGTA, 10 mM β-glycerophosphate, 2 mM Na3VO4, 1 mM NaF, 1 mM DTT, cocktail protease inhibitor plus RNase inhibitor). Solubilization of crosslinked complexes was done by mechanical sonication by three rounds of sonication, 20s each, in a Microson XL2007 ultrasonic homogenizer with a microprobe at an amplitude setting of 7 (output, 8–9 W). Insoluble materials were removed by microcentrifugation at 14,000 rpm (16,000g) for 20 min at 4°C. Take 10% out of Input. Preclearing lysates with 20 µl of PBS washed Magna CHIP Protein A+G Magnetic Beads (Millipore, Massachusetts). The precleared lysate (250 µl) was then diluted with dilution buffer (250 µl), mixed with the specific antibody-coated beads, and incubated with rotation at 4°C for 4h, followed by 4 times washing with Low Salt buffer 1 time, High Salt Buffer 1 time, LiCl 1 time, and TE buffer 2 times containing protease inhibitors. The beads were eluted with 0.1M NaHCO3 Elution Buffer at room temperature for 20mins and the formaldehyde cross-links was reversed at 65°C overnight. The collected immunoprecipitated complexes and input were digested in protein digestion Buffer with 10 µg Proteainse K and incubated at 45 °C for 1 hour. DNA was extracted from these samples using phenol/ chloroform according to the manufacturer’s protocol (Invitrogen Corp.). The presence of DNA was measured by quantitative, strand-specific RT-PCR using the iCycler iQ Real-time detection system (Bio-Rad). Gene-specific PCR primer pairs are listed in S3 Table. The following antibodies were used for ChIP analysis: anti-p65, anti-p50, anti-HDAC1, anti-H3K9me3, and anti-H3K27me3 (Cell Signaling Technology).

**Bioinformatics Analyses**

After sequencing, the raw reads were filtered. Data filtering includes removing adaptor sequences, contamination and low-quality reads from raw reads. Next, we get the statistics of data production. Supplemental Table 4 shows statistical results of m^6^A-RIP-Seq sequence.

For RNA-Seq analysis, the low-quality reads, which have more than 20% bases with a quality score smaller than 10 or more than 5% of ambiguity bases (N), were filtered using SOAPnuke v1.5.2 (Chen et al., 2018). The clean reads were mapped using HISAT2 v2.0.4 (Kim et al. 2015). The program StringTie v1.0.4 (Pertea et al., 2015) was used to reconstruct transcripts and the Cuffcompare in Cufflinks v2.2.1 (Tranell et al., 2012) was used to compare reconstructed transcripts to reference annotation. The prediction of novel transcripts was conducted using CPC v0.9-r2 (Kong et al., 2007). The complete reference transcriptome was constructed by merging the novel transcripts with reference transcripts and used for downstream analysis. The clean reads were then mapped to the constructed reference transcriptome using Bowtie2 v2.2.5 (Langmead et al., 2012) and the gene expression level was calculated with RSEM (Li and Dewey 2011).

For m^6^A-RIP-Seq analysis, after removing the adapter and low-quality reads, the reads were mapped to mouse genome (mm10) and RefSeq mRNA sequences (ftp://hgdownload.cse.ucsc.edu/goldenPath/mm10/bigZips/refMrna.fa uncompressed) using Bowtie2 v2.2.5 (Langmead et al., 2012). The m^6^A peaks were detected using MACS2 with each pair of mapped files (infested versus control) as input (Feng et al., 2012). The peaks were called if a fold change was more than 5 and individual FDR value was smaller than 5%. The detection of m^6^A methylation sites with significant alterations in infested versus control groups using DiffBind v2.10.0 was used (Ross-Innes et al., 2012). The DiffBind analysis involves five phases, including reading in peak sets, occupancy analysis, counting reads, differential binding affinity analysis, and reporting. The differential analysis of m^6^A methylations was executed using DESeq2 (Love et al., 2014), which assigns a p-value and an FDR to each candidate region, indicating confidence of the change of m^6^A methylation. The annotation and visualization of m^6^A mthylation sites, i.e., promoter, UTR and CDS were performed using ChIPseeker v1.18.0 (Yu et al., 2015). The sequences in these modification regions were extracted using bedtools GetFastaBed (Quinlan and Hall 2010) from the RefSeq mRNA sequences with the intervals identified by DiffBind. Motif discovery, enrichment analysis and clustering were conducted by using MEME 4.11.2 (Machanick and Bailey 2011). MEME uses several statistical modeling methods, log likelihood ratio, information content, relative entropy, and Bayes threshold to find the best width, number of occurrences, and description for each motif (Machanick and Bailey 2011). Gene Ontology (GO) classifications were analyzed using the PANTHER system (Mi et al., 2012). Correlation and related statistical tests were conducted in R v3.6.3.

**References**

1. Chen, Y., Chen, Y., Shi, C., Huang, Z., Zhang, Y., Li, S., ... & Zhang, X. (2018). SOAPnuke: a MapReduce acceleration-supported software for integrated quality control and preprocessing of high-throughput sequencing data. Gigascience, 7(1), gix120.
2. Feng, Jianxing and Liu, Tao and Qin, Bo and Zhang, Yong and Liu, Xiaole Shirley (2012). Identifying ChIP-seq enrichment using MACS. In Nature Protocols, 7 (9), pp. 1728-1740. [doi:10.1038/nprot.2012.101]
3. Kim, D., Langmead, B. & Salzberg, S. L. (2015). HISAT: a fast spliced aligner with low memory requirements. Nat. Methods 12, 357-360.
4. Kong, L. et al. (2007). CPC: assess the protein-coding potential of transcripts using sequence features and support vector machine. Nucleic Acids Res. 35, W345-W349.
5. Hu, G., A. Y. Gong, Y. Wang, S. Ma, X. Chen, J. Chen, C. J. Su, A. Shibata, J. K. Strauss-Soukup, K. M. Drescher, et al. 2016. LincRNA-Cox2 promotes late inflammatory gene transcription in macrophages through modulating SWI/SNF-mediated chromatin remodeling. *J. Immunol.* 196: 2799–2808.
6. Langmead, B. et al. (2012). Fast gapped-read alignment with Bowtie 2. Nat. Methods 9, 357-359.
7. Li, B. & Dewey, C. N. (2011). RSEM: accurate transcript quantification from RNA-Seq data with or without a reference genome. BMC Bioinformatics 12, 323.
8. Love, M. I., Huber, W., & Anders, S. (2014). Moderated estimation of fold change and dispersion for RNA-seq data with DESeq2. Genome biology, 15(12), 550.
9. Machanick, P., & Bailey, T. L. (2011). MEME-ChIP: motif analysis of large DNA datasets. *Bioinformatics*, *27*(12), 1696-1697.
10. Mi, H., Muruganujan, A., & Thomas, P. D. (2012). PANTHER in 2013: modeling the evolution of gene function, and other gene attributes, in the context of phylogenetic trees. Nucleic acids research, 41(D1), D377-D386.
11. Pertea, M. et al. (2015). StringTie enables improved reconstruction of a transcriptome from RNA-seq reads. Nat. Biotechnol. 33, 290-295.
12. Quinlan, Aaron R. and Hall, Ira M. (2010). BEDTools: a flexible suite of utilities for comparing genomic features. Bioinformatics, 26 (6), pp. 841-842.
13. R Core Team (2013). R: A language and environment for statistical computing. R Foundation for Statistical Computing, Vienna, Austria.
14. Ross-Innes, C. S., Stark, R., Teschendorff, A. E., Holmes, K. A., Ali, H. R., Dunning, M. J., ... & Ali, S. (2012). Differential oestrogen receptor binding is associated with clinical outcome in breast cancer. *Nature*, *481*(7381), 389-393.
15. Tong, Q., A. Y. Gong, X. Zhang, C. Lin, S. Ma, J. Chen, G. Hu, and X. M. Chen. 2016. LincRNA-Cox2 modulates TNFα-induced transcription of *Il12b* gene in intestinal epithelial cells through regulation of Mi-2/NuRD-mediated epigenetic histone modifications. *FASEB J.* 30: 1187–1197.
16. Trapnell, C., Roberts, A., Goff, L., Pertea, G., Kim, D., Kelley, D. R., ... & Pachter, L. (2012). Differential gene and transcript expression analysis of RNA-seq experiments with TopHat and Cufflinks. *Nature protocols*, *7*(3), 562-578.
17. Yu, Guangchuang and Wang, Li-Gen and He, Qing-Yu (2015). ChIPseeker: an R/Bioconductor package for ChIP peak annotation, comparison and visualization. Bioinformatics, 31 (14), pp. 2382-2383.
